# Zinc Exposure Promotes Commensal-to-Pathogen Transition in *Pseudomonas aeruginosa* Leading to Mucosal Inflammation and Illness in Mice

**DOI:** 10.3390/ijms222413321

**Published:** 2021-12-11

**Authors:** Tong Wu, Annie Gagnon, Katherine McGourty, Rebecca DosSantos, Lucia Chanetsa, Boce Zhang, Dhimiter Bello, Shannon L. Kelleher

**Affiliations:** Department of Biomedical and Nutritional Sciences, Zuckerberg College of Health Sciences, University of Massachusetts Lowell, 883 Broadway Street, Dugan Hall 110R, Lowell, MA 01852, USA; tong_wu@student.uml.edu (T.W.); gagnona95@gmail.com (A.G.); mcgourtykatie1@gmail.com (K.M.); rebecca_dossantos@student.uml.edu (R.D.); lucia_chanesta@uml.edu (L.C.); boce_zhang@uml.edu (B.Z.); Dhimiter_Bello@uml.edu (D.B.)

**Keywords:** zinc, *Pseudomonas aeruginosa*, virulence, Caco-2 cells, intestinal permeability, colonic inflammation

## Abstract

The opportunistic pathogen *Pseudomonas aeruginosa* (*P. aeruginosa*) is associated gastrointestinal (GI) inflammation and illness; however, factors motivating commensal-to-pathogen transition are unclear. Excessive zinc intake from supplements is common in humans. Due to the fact that zinc exposure enhances *P. aeruginosa* colonization in vitro, we hypothesized zinc exposure broadly activates virulence mechanisms, leading to inflammation and illness. *P. aeruginosa* was treated with excess zinc and growth, expression and secretion of key virulence factors, and biofilm production were determined. Effects on invasion, barrier function, and cytotoxicity were evaluated in Caco-2 cells co-cultured with *P. aeruginosa* pre-treated with zinc. Effects on colonization, mucosal pathology, inflammation, and illness were evaluated in mice infected with *P. aeruginosa* pre-treated with zinc. We found the expression and secretion of key virulence factors involved in quorum sensing (QS), motility (type IV pili, flagella), biosurfactants (rhamnolipids), toxins (exotoxin A), zinc homeostasis (CzcR), and biofilm production, were all significantly increased. Zinc exposure significantly increased *P. aeruginosa* invasion, permeability and cytotoxicity in Caco-2 cells, and enhanced colonization, inflammation, mucosal damage, and illness in mice. Excess zinc exposure has broad effects on key virulence mechanisms promoting commensal-to-pathogen transition of *P. aeruginosa* and illness in mice, suggesting excess zinc intake may have adverse effects on GI health in humans.

## 1. Introduction

*Pseudomonas aeruginosa* (*P. aeruginosa*) is a commensal Gram-negative opportunistic pathogen in the GI tract [1]. The association between *P. aeruginosa* and GI disorders is often underestimated; however, an increasing number of studies connect *P. aeruginosa* colonization to GI disease development. For example, the gut microbiome of children with Crohn’s disease (CD) is significantly enriched in *Pseudomonas* spp. [2]. In addition, *Pseudomonas* spp. were found in 11% of adult CD and ulcerative colitis (UC) patients, while not detected in healthy adults [3]. Additionally, *Pseudomonas* spp. was detected in 8.3% of patients with irritable bowel syndrome (IBS), but only 0.1% of healthy subjects [4]. Moreover, *P. aeruginosa* has been specifically associated with several diarrheal diseases, such as Shanghai fever, pseudomonal enterocolitis, and antibiotic-associated diarrhea [5], and is a common cause of antibiotic-associated diarrhea in immunocompromised patients [6]. Importantly, *P. aeruginosa* has been isolated from the blood of the patients suffering neutropenic enterocolitis [7], illustrating its ability to breach the GI barrier leading to systemic infection. However, host-associated factors that drive the commensal-to-pathogen shift are not understood.

Numerous *P. aeruginosa* virulence factors are responsible for infection and inflammation in the GI tract [8]. The *las* quorum sensing (QS) system is governed by *lasR* and *lasI*, which direct the synthesis of the extracellular autoinducer N-(3-Oxododecanoyl)-l-homoserine lactone (3-Oxo-C12HSL) [9]. 3-Oxo-C12HSL suppresses the expression of E-cadherin and β-catenin, leading to the disruption of tight junction structures and greater intestinal permeability [10]. In addition, *P. aeruginosa* motility is primarily mediated by the surface-exposed fibers, type IV pili and flagella [11,12]. *P. aeruginosa* relies on flagella to form a single cell layer on the epithelial surface, which then aggregates into microcolonies by the twitching movement of type IV pili, forming a biofilm on the host epithelium and leading to a more persistent GI infection [13,14]. Flagellin is also recognized by a toll-like receptor (TLR) 5 and activates mucosal inflammatory pathways [15]. Once internalized, *P. aeruginosa* releases flagellin, which binds to the cytoplasmic domain of TLR5 and activates the myeloid differentiation 88 (MyD88) and nuclear factor κ-beta (NF-κB) pathways, leading to induction of interleukin (IL) -1β and IL-6 [16,17]. Moreover, *P. aeruginosa* produces and secretes rhamnolipids, a biosurfactant composed of hydrophobic fatty acids and hydrophilic rhamnose [18]. Rhamnolipids have significant antimicrobial properties against other bacteria such as *Serratia marcescens*, *Enterobacter aerogenes*, *Klebsiella pneumoniae*, *Staphylococcus aureus* and *Listeria monocytogenes*, and fungi such as *Fusarium solani* and *Penicillium funiculosum* [19,20]. With this antimicrobial ability, rhamnolipids provide *P. aeruginosa* with a significant advantage in competing and eliminating other bacteria and fungi, promoting its enrichment in the GI tract. Furthermore, as the most lethal component secreted by *P. aeruginosa*, exotoxin A is an extracellular enzyme, which once internalized inhibits protein synthesis and causes necrosis of intestinal tissues [21], indirectly causing host inflammatory responses. Stressed or necrotic intestinal tissue initiates release of intracellular substances, such as nuclear and mitochondrial DNA, which bind to TLR9 or nucleotide-binding oligomerization domain (NOD)-like receptors, leading to the production of a broad range of pro-inflammatory cytokines [22,23]. Thus, once virulence pathways are activated, *P. aeruginosa* becomes a formidable bacterial pathogen; however, the luminal factors in the environment of the GI tract that activate commensal-to-pathogen transition are not understood.

Zinc is an essential mineral [24]; the estimated average requirement (EAR) is 11 mg/d for men and 8 mg/d for women. In addition to dietary consumption, ~50% of US adults consume supplements generally containing 5–50 mg of zinc [25]. This leads to excessive levels of zinc intake that well exceed recommendations in a large percentage of the US population; in fact, >10% of US adults consume zinc levels above the Tolerable Upper Limit (40 mg/d) [26]. While modest zinc supplementation (5–10 mg zinc/d) is used therapeutically to reduce clinical symptoms of IBS, inflammatory bowel disease (IBD), and diarrhea [27], excess zinc consumption, particularly in otherwise healthy individuals, may be associated with increased risk for GI disorders [28]. For example, excess zinc (250 µM) alters the morphology of cultured intestinal epithelial cells and increases permeability [29]. Excessive zinc consumption in mice (100 mg zinc/kg diet) leads to mucosal inflammation and epithelial damage, and leaves mice more susceptible to *Clostridium difficile* infection in the GI tract [30]. In addition, excess zinc has serious indirect consequences on gut function through alterations in the microbiome. When chronically fed to piglets, excess zinc (~2000 mg zinc/kg diet) leads to the broad induction of zinc-tolerant bacterial strains [31], and colonization of multi-antibiotic resistant *Enterobacteriaceae* [32]. In vitro studies demonstrate excess zinc (>60 µM) enhances the pathological effects of bacterial endotoxins such as lipopolysaccharide (LPS), potentiating the production of NF-κB leading to broad inflammatory responses [33]. Moreover, we previously reported that excessive zinc consumption in mice (100 µg zinc/d) enriches the gut colonization of *Enterobacteriaceae*, *Clostridia*, *Bacteroides*, *Campylobacter* and *Pseudomonadales* spp., and the presence of *P. aeruginosa* was specifically identified [34]. However, studies exploring effects of excess zinc on *Pseudomonas* are sparse and conflicting. While ZnCl_2_ (200–1000 µM) induces biofilm formation in *P. aeruginosa* [35], high concentrations of zinc oxide (1000 µM) inhibit *P. aeruginosa* growth and reduce biofilm formation [36] and zinc oxide nanoparticles (>4 mM) inhibit *P. aeruginosa* virulence by inhibiting QS signaling via *lasR*-*rhlR* suppression [37,38]. This underscores the importance of understanding the effects of an excessive intake of a pharmacologically relevant zinc source on the commensal-to-pathogen transition of *P. aeruginosa* and the susceptibility to GI inflammation.

By using *P. aeruginosa* as a representative opportunistic nosocomial gut pathogen and zinc sulfate (ZnSO_4_) as one of the most common forms of zinc supplementation, we sought to test the hypothesis that excess dietary zinc exposure promotes a commensal-to-pathogen transition by upregulating the expression of key virulence factors, and that these changes enhance the inflammation and necrosis in intestinal epithelial cells, leading to increased mucosal inflammation and illness. Here we conducted molecular studies to understand the effects of excess zinc exposure on the key virulent factors in *P. aeruginosa* that are involved in the commensal-to pathogen transition in vitro, investigated cytotoxic effects of zinc-activated virulence using a co-culture model in human intestinal cells, and explored the physiological relevance of zinc-induced virulence on GI function and illness in a pre-clinical mouse model.

## 2. Results

### 2.1. Effect of Zinc on P. aeruginosa Growth

**Figure 1:**
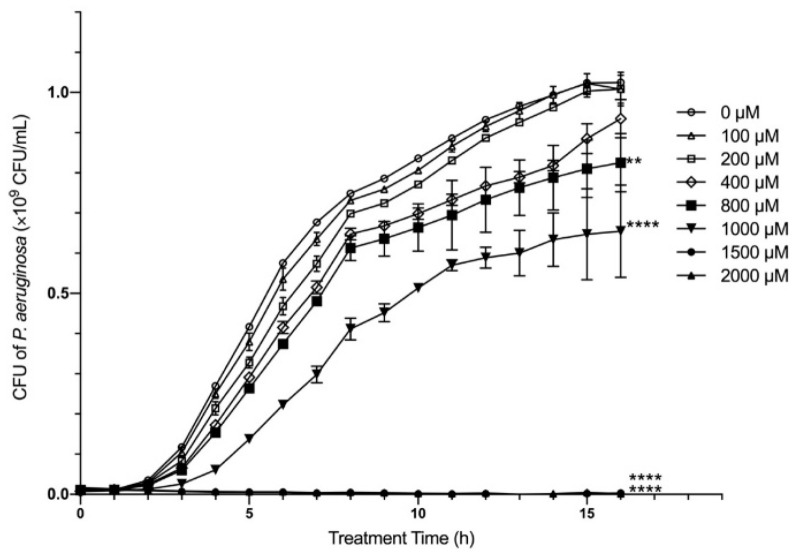
The effect of zinc on *P. aeruginosa* growth. In vitro growth of PAO1 in the presence of ZnSO_4_ (0–2000 µM) was measured every hour for 16 h. The data shown are from one representative experiment, using three independent samples/groups. The data represent the mean CFU × 10^9^/mL of PAO1 ± SD. ** *p* < 0.01, **** *p* < 0.0001 using Kruskal–Wallis. The experiment was repeated twice.

For these studies we chose to use ZnSO_4_ as it is one of the most common forms of zinc supplement; however, the amount of zinc in dietary supplements varies widely, ranging from 5–50 mg. Previous studies exploring effects of zinc on *P. aeruginosa* in vitro used a wide range in zinc concentration (200–4000 µM) [35,37,38]. However, studies in humans show consumption of 5–10 mg zinc leads to an intraluminal concentration of ~375–950 µM [39,40]. Therefore, we limited the range of zinc concentrations in this study (100–2000 µM). We first tested the effects on growth, by measuring the colony forming units (CFU) of *P. aeruginosa* PAO1 (PAO1), the most commonly used reference strain, following 16 h zinc exposure. As shown in Figure 1, >1500 µM completely eliminated growth (*p* < 0.0001), while 1000 µM significantly reduced growth (*p* < 0.0001). In contrast, 800 µM and 400 µM had more modest effects on PAO1 growth (*p* < 0.01), while zinc concentrations < 400 µM had no effect. Since we determined 800 µM was physiologically feasible following low dose zinc supplementation, that this concentration was in the middle of the range used in comparative studies, and that this had a profound effect on PAO1 growth, we used 800 µM as the zinc concentration for the remaining studies.

### 2.2. Zinc Increased Key Virulent Factors in P. aeruginosa In Vitro

**Figure 2:**
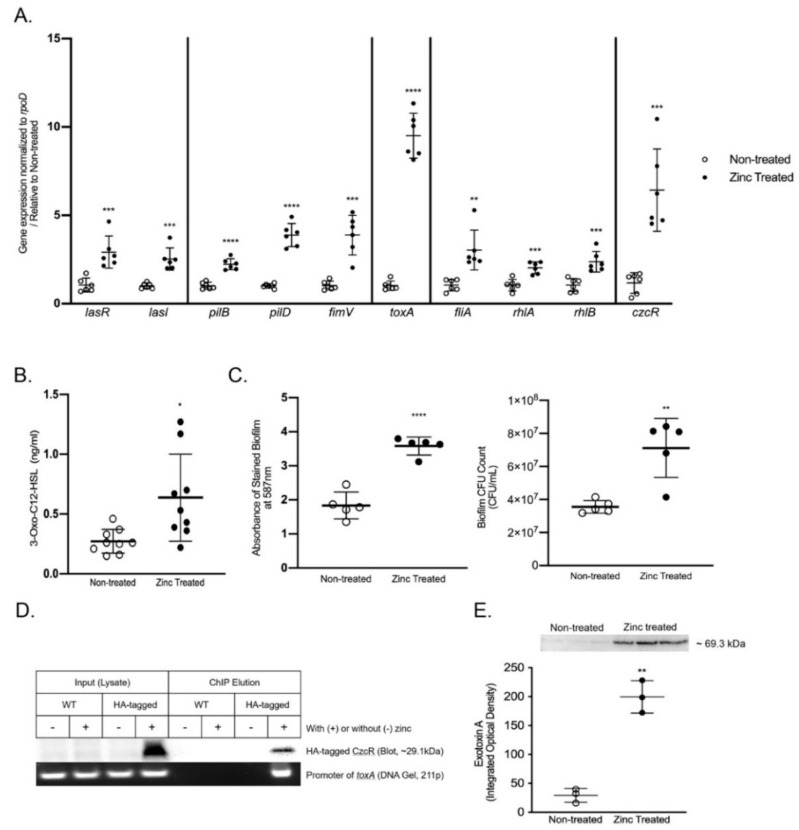
The effect of zinc on *P. aeruginosa* virulence. PAO1 was exposed to ZnSO_4_ (800 µM) for 16 h (zinc treated) and effects on key virulence pathways were compared to *P. aeruginosa* that was not exposed to excess zinc (non-treated). (**A**) Virulence gene expression. The data shown were generated from two independent experiments, using three independent samples/groups in each experiment. The data represent mean gene expression relative to rpoD ± SD, normalized to non-treated PAO1. *** *p* < 0.001, **** *p* < 0.0001 by student *t* test or ** *p* < 0.01 by Mann–Whitney. (**B**) Quantitation of 3-Oxo-C12 HSL in conditioned medium. The data shown were generated from three independent experiments, using three independent samples/groups in each experiment. The data represent the mean concentration of 3-Oxo-C12 HSL (ng/mL) ± SD. * *p* < 0.05 by student *t* test. (**C**) Biofilm formation. The data shown are from one representative experiment, using five independent samples/groups. The data represent the mean absorbance at 587 nm ± SD and the mean CFU/mL ± SD. ** *p* < 0.01, **** *p* < 0.0001 by student *t* test. The experiment was repeated twice. (**D**) Chromatin immunoprecipitation. Representative images of an immunoblot of hemagglutinin (HA; top) and an ethidium bromide-stained agarose gel (bottom). After verifying that HA-tagged CzcR protein was successfully expressed (Input Lysate; HA-tagged +) and precipitated by immunoblotting (ChIP elution; HA-tagged +), PCR was used to amplify the promoter region of toxA in CzcR-HA PAO1 treated for 16 h with zinc. Zinc-treated (WT; +) and non-treated PAO1 (WT; −), and non-zinc treated CzcR-HA PAO1 (HA-tagged; −) were used as negative controls. The ChIP experiment was repeated three times, using three independent samples. (**E**) Quantification of exotoxin A in the conditioned medium. Representative immunoblot of exotoxin A detected in the conditioned medium from PAO1 treated with (zinc treated) and without (non-treated) zinc. The data shown are from one representative experiment, using three independent samples/groups. The data represent the mean integrated optical density ± SD. ** *p* < 0.01 by student *t* test. The experiment was repeated twice.

To systematically determine the effects of zinc on the commensal-to-pathogen transition in *P. aeruginosa*, we selected a broad series of key virulence genes to evaluate that included factors associated with the QS system, motility regulation, rhamnolipids, and exotoxins (Figure 2). To assess the effects of zinc exposure on the activation of the QS system, we measured lasR and lasI gene expression using RT-qPCR. Zinc exposure significantly increased the expression in both lasR and lasI (*p* < 0.001) by ~3-fold (Figure 2A). As the expression of lasR and lasI were increased, we also quantified the amount of their signaling molecules, 3-Oxo-C12HSL and C4-HSL, secreted into the culture medium using LC-ESI-MS/MS. C4-HSL was not detected; however, the concentration of 3-Oxo-C12HSL was significantly greater in the culture medium collected from zinc-treated PAO1 compared with non-treated PAO1 (*p* < 0.05, Figure 2B). To evaluate the effects of zinc exposure on the activation of motility and biofilm formation, we determined the effects of zinc exposure on the gene expression of type IV pili genes (pilB/D and fimV) by RT-qPCR. As shown in Figure 2A, zinc exposure significantly increased pilB and pilD expression (*p* < 0.0001) by ~3-fold and ~4-fold, respectively, and fimV by ~4-fold (*p* < 0.001). In order to confirm that the zinc-dependent increases in pilB/pilD/fimV expression were associated with greater biofilm production, we measured the biofilm formation directly using crystal violet staining and CFU counting. As shown in Figure 2C, zinc exposure significantly increased biofilm production (*p* < 0.0001) and led to significantly greater colony formation within the biofilm (*p* < 0.01). The effects of zinc exposure on motility and rhamnolipids production were determined by measuring the gene expression of fliA (major flagella regulator) and rhlA/B (Rhamnolipids rhamnosyl-transferase) by RT-qPCR. As shown in Figure 2A, zinc exposure significantly increased fliA, rhlA and rhlB expression ~2-fold (*p* < 0.01, *p* < 0.001, *p* < 0.001, respectively).

To evaluate the effects of zinc exposure on exotoxin production, we first determined the effects on toxA gene expression. As shown in Figure 2A, toxA expression was profoundly upregulated (~10-fold, *p* < 0.0001) by zinc, suggesting excess zinc may have a direct effect on toxA expression. CzcR is the only known transcriptional regulator activated by excess zinc [41]. In addition, CzcR co-regulates the virulence factor lasI in *P. aeruginosa* in response to excess zinc exposure [41]. Therefore, we hypothesized that CzcR co-regulates the expression of toxA. As shown in Figure 2A, we confirmed that the expression of czcR was upregulated by excess zinc exposure (~7-fold, *p* < 0.001). Moreover, we conducted chromatin immunoprecipitation (ChIP) assays to determine whether the effect of excess zinc exposure on toxA expression was indeed driven by CzcR. PAO1 expressing CzcR-HA was treated with excess zinc (800 µM) to activate czcR expression, and CzcR was cross-linked to DNA. DNA was sheared by sonication and soluble protein was collected. DNA bound to CzcR was immunoprecipitated, and DNA-protein interaction was assessed by immunoblotting and PCR. Zinc-treated and non-treated PAO1, and non-treated CzcR-HA expressing PAO1 were used as controls. Importantly, we determined that as was reported for lasI, zinc exposure promoted CzcR binding to the toxA promoter (Figure 2D). We next used immunoblotting to confirm increased production and secretion of exotoxin A in the culture medium collected from zinc-treated PAO1. As shown in Figure 2E, exotoxin A was barely detectable in the culture medium collected from non-treated PAO1. However, after zinc exposure, exotoxin A was profoundly enriched (*p* < 0.01). Collectively, these data confirm that excess zinc exposure in *P. aeruginosa* promotes commensal-to-pathogen transition and suggests that CzcR-mediated transcriptional regulation may be a universal virulence mechanism activated by zinc exposure.

### 2.3. Zinc-Exposure Enhanced P. aeruginosa Virulence in Caco-2 Cells

**Figure 3:**
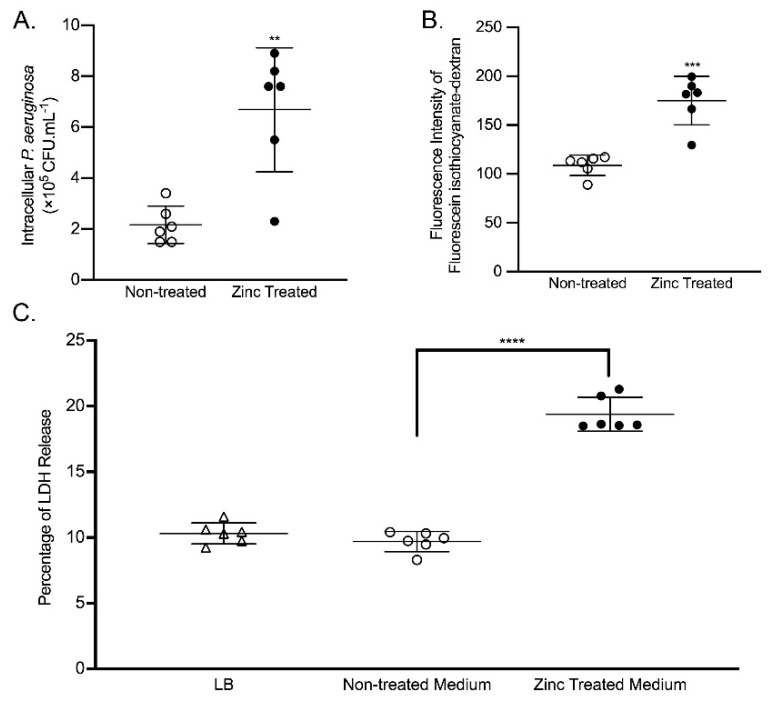
The effect of zinc-exposed *P. aeruginosa* on Caco-2 cells. (**A**) Zinc-exposed *P. aeruginosa* increased invasive capacity. Caco-2 cells were co-cultured for 4 h with PAO1 previously exposed to ZnSO_4_ (800 µM) for 16 h (zinc treated) or non-treated PAO1, and extracellular bacteria were killed by gentamicin. Caco-2 cells were lysed, and the intracellular bacteria were enumerated by plating onto LB Agar. The data shown were generated from two independent experiments, using three independent samples/groups. The data represent the mean CFU × 10^5^/mL ± SD. ** *p* < 0.01 by student *t*-test. (**B**) Zinc-exposed *P. aeruginosa* increased Caco-2 cell monolayer permeability. Caco-2 cells were co-cultured for 12 h with PAO1 previously exposed to ZnSO_4_ (800 µM) for 16 h (zinc treated) or non-treated PAO1. Fluorescein isothiocyanate-dextran was added to the apical chamber. The medium in the basolateral chamber was collected, and the fluorescence intensity was measured. The data shown were generated from two independent experiments, using three independent samples/groups. The data represent the mean fluorescence intensity ± SD. *** *p* < 0.001 by student *t*-test. (**C**) Zinc-exposed *P. aeruginosa* increased cell death. Caco-2 cells were co-cultured for 6 h with conditioned medium from PAO1 previously exposed to ZnSO4 (800 µM) for 16 h (Zinc treated medium) or non-treated PAO1 medium. Caco-2 cells co-cultured with fresh LB for 6 h were used as a negative control. Cytotoxicity was determined by LDH assay. The data shown were generated from two independent experiments, using three independent samples/groups. The data represent the mean percentage of LDH released relative to total LDH ± SD. **** *p* < 0.0001 by ANOVA.

To evaluate the consequence of excess zinc-exposed *P. aeruginosa* on intestinal cell function, we pre-treated PAO1 with excess zinc (800 µM) for 16 h. Bacteria were collected, washed and resuspended in fresh Dulbecco’s Modified Eagle’s Medium (DMEM, without serum and antibiotics). A post-confluent monolayer of Caco-2 cells was co-cultured with either zinc-treated or non-treated PAO1 for 4 h, and Caco-2 cells were then incubated with 300 µg/mL gentamycin (GM) to kill any remaining extracellular PAO1. Cells were lysed and the cell lysates were plated on agar plates before being incubated at 37 °C overnight, and viable PAO1 that were internalized were quantified by plate-counting. As shown in Figure 3A, excess zinc exposure significantly increased the number of PAO1 that were able to invade Caco-2 cells (~4-fold, *p* < 0.01).

To determine the effects of excess zinc-exposed *P. aeruginosa* on intestinal permeability, we measured cellular permeability to fluorescein isothiocyanate-dextran on post-confluent Caco-2 monolayers infected with zinc-exposed PAO1. As shown in Figure 3B, Caco-2 cells co-cultured with zinc-exposed PAO1 had significantly higher (~1.5-fold, *p* < 0.001) fluorescence intensity in the medium in the basolateral chamber compared to cells co-cultured with non-exposed PAO1, suggesting that excess zinc exposure enhances the ability of *P. aeruginosa* to disrupt the intestinal epithelium and increase intestinal permeability.

Exotoxin A is the most cytotoxic compound produced by *P. aeruginosa*. To confirm that exotoxin A produced in response to excess zinc exposure was biologically competent, we treated Caco-2 cells with conditioned and filtered (to remove any residual bacteria) medium from zinc-exposed PAO1 and measured the amount of lactate dehydrogenase (LDH) released as an index of cell death. As shown in Figure 3C, the culture medium containing exotoxin A from zinc-exposed PAO1 generated significantly greater cytotoxicity compared to the culture medium from PAO1 not exposed to excess zinc (~2-fold, *p* < 0.0001). Importantly, the conditioned medium from PAO1 not exposed to excess zinc had no effect on LDH release, which is consistent with its lack of pathogenicity. This provides evidence that excess zinc exposure drives the commensal-to-pathogen transition and leads to *P. aeruginosa*-mediated cytotoxicity in the intestinal epithelium.

### 2.4. Zinc-Induced Virulence Causes GI Dysfunction and Illness In Vivo

**Figure 4:**
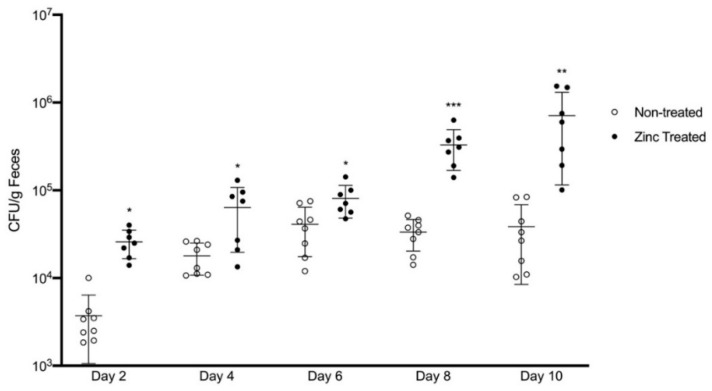
Zinc treatment increased the ability of *P. aeruginosa* to colonize the mouse GI tract. PAO1 was exposed to ZnSO_4_ (800 µM) for 16 h (Zinc treated) or left untreated (non-treated) and orally gavaged in mice. Colony forming units (CFU) from the feces of mice inoculated with zinc treated or non-treated PAO1 were quantified every two days by plating the feces on *P. aeruginosa*-selective cetrimide agar plates. The data represent the mean CFU/g feces ± SD, *n* = 7–8 mice/group. * *p* < 0.05, ** *p* < 0.01, *** *p* < 0.001, relative to non-treated PAO1 mice at each timepoint, by student *t*-test.

With this result, we next determined the impact of excess zinc-mediated commensal-to-pathogen transition on gut pathology and illness in a preclinical mouse model. To isolate the effects of excess zinc exposure on *P. aeruginosa* virulence from the effects of excess zinc on the host GI tract, mice were fed a diet containing a normal amount of zinc (25 mg zinc/kg/diet) for 2 weeks prior to being orally gavaged with PAO1 that had been pre-treated with zinc (800 µM) or left untreated. As noted previously, *P. aeruginosa* is normally a minor commensal gut bacterium, and enrichment in the gut environment is the first pathoadaptive trait. To confirm that *P. aeruginosa* was able to colonize the mouse GI tract, feces were collected, fecal extracts were generated, and CFU counting was performed. As shown in Figure 4, *P. aeruginosa* successfully colonized both groups of mice. However, while colonization of non-treated PAO1 was detected at d2 and stabilized at ~5 × 10^4^ CFU/g feces after d6, zinc-exposed PAO1 had significantly greater colonization by d2 (*p* < 0.05), colonization remained significantly greater compared to non-treated PAO1 throughout the study, and colonization continued to increase throughout the entire study period. By the end of the study at d10, colonization of zinc-exposed PAO1 was ~18-fold greater (7.1 × 10^5^ CFU/g feces) compared to mice colonized with non-treated PAO1 (3.9 × 10^4^ CFU/g feces; *p* < 0.01). It is critical to note that the mice themselves were not exposed to excess dietary zinc, thereby indicating that the enhanced colonization resulted from a direct effect of the excess zinc activating virulence mechanisms in *P. aeruginosa*.

**Figure 5:**
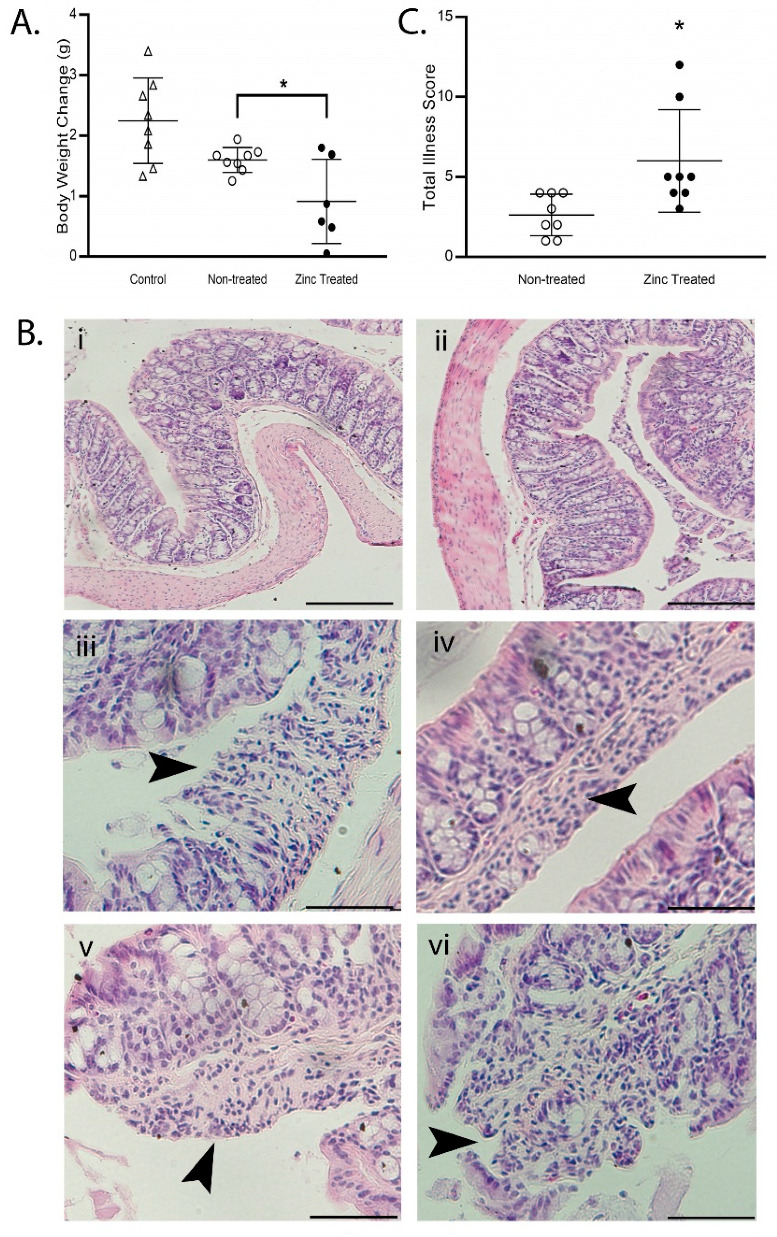
Infection with zinc-exposed *P. aeruginosa* causes illness in mice. PAO1 was exposed to ZnSO_4_ (800 µM) for 16 h (zinc treated) or left untreated (non-treated) and orally gavaged in mice who were fed a control diet for 2 weeks prior to oral gavage. Mice not gavaged with PAO1 were used for comparison (control). (**A**) Zinc-exposed *P. aeruginosa* decreased body weight gain. The data represent the mean body weight change (g) ± SD, *n* = 6–8 mice/group. * *p* < 0.05, relative to non-treated PAO1 mice, by ANOVA. Note that infection with non-treated PAO1 did not significantly decrease body weight. (**B**) Zinc-exposed *P. aeruginosa* caused colonic pathology. Representative images of H&E-stained colon sections. Note that the histology in control mice (**i**) and mice infected with non-treated PAO1 (**ii**) was similar and had no pathological manifestations. Architectural distortion was found only in colons of mice infected with zinc treated PAO1 (**iii**–**vi**). Arrowheads identify classical histopathology including effacement (**iii**), neutrophil infiltration (**iv**), and ulcer formation (**v**,**vi**). Magnification, 10× for control and non-treated PAO1 images (scale bar, 100 µm); 40× for zinc treated PAO1 images to highlight architectural distortion (scale bar, 50 µm). (**C**) Zinc-exposed *P. aeruginosa* increased total illness score. Illness was scored using the following parameters: daily body weight loss; general behavior; stool consistency; stool blood; and colon epithelial morphology and inflammatory cell infiltration documented using H&E staining [42]. The data represent the mean total illness score ± SD, *n* = 8 mice/group. * *p* < 0.05 by student *t*-test.

We first determined pathophysiological consequences of the GI enrichment of zinc-exposed *P. aeruginosa*. As shown in Figure 5A, at 10 d post-inoculation, the mean weight gain of mice infected with non-treated PAO1 was similar to that of non-infected mice. In contrast, mice infected with zinc-exposed PAO1 had significantly lower mean weight gain when compared with both the mice infected with non-treated PAO1 and the non-infected mice (*p* < 0.05). Moreover, to determine the effects on the colon directly, we stained the colon with hematoxylin and eosin (H&E) and scored the histological findings. As shown in Figure 5B, architectural distortion, including brush border effacement and ulcer formation, was detected in the colons of 5/8 mice infected with zinc-exposed PAO1. However, these defects were not observed in any mice that were infected with non-treated PAO1. In addition, mice infected with zinc-exposed PAO1 had a significantly higher overall illness score than mice infected with non-treated PAO1 (*p* < 0.05, Figure 5C). This provides compelling evidence that excess zinc exposure enhances the commensal-to-pathogen transition in *P. aeruginosa* and causes intestinal pathology and illness.

**Figure 6:**
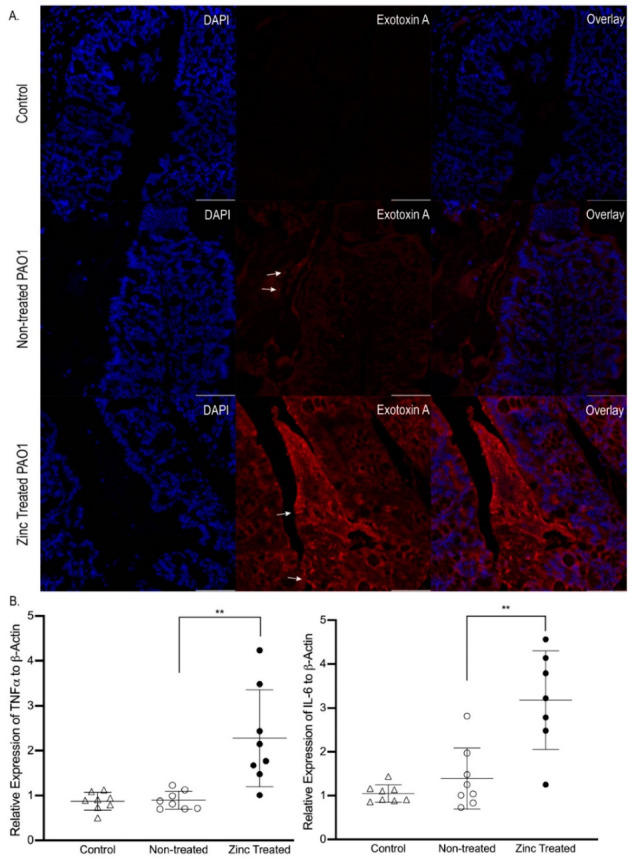
Infection with zinc-exposed *P. aeruginosa* induced greater mucosal inflammatory responses in mice. PAO1 was exposed to ZnSO_4_ (800 µM) for 16 h (zinc treated) or left untreated (non-treated) and orally gavaged in mice. Mice not gavaged with PAO1 were used for comparison (control). (**A**) Mice infected with zinc-exposed *P. aeruginosa* had greater exotoxin A in the colon. Representative images of exotoxin A (red) and nuclei stained with DAPI (blue) in colon of control mice, and mice infected with Non-treated PAO1 or Zinc-treated PAO1. No exotoxin A was observed in control mice (negative control). In mice infected with Non-treated PAO1, little fluorescence was detected, and what was detected was observed as punctate rods, indicative of intact PAO1 (white arrows). In contrast, intense fluorescence was detected as both an overall haze throughout the tissue and as punctate rods (white arrows) in mice infected with Zinc treated PAO1. Additionally, the formation of biofilm structures was only detected in the colons of mice infected with Zinc treated PAO1 (note defined triangular structure between colonic folds). Magnification, 40×; Scale bar, 50 µm. (**B**) Zinc-exposed *P. aeruginosa* activated colon inflammation. Data represent mean fold change of TNFα, and IL-6 mRNA levels normalized to β-actin ± SD, relative to control mice, *n* = 7–8 mice/group. ** *p* < 0.01 by ANOVA. Note that infection with Non-treated PAO1 caused no increase in TNFα and IL-6 mRNA levels.

Since we showed the excess zinc-exposure increased the expression and secretion of exotoxin A, we used immunofluorescence (IF) imaging to detect exotoxin A in the colon. As shown in Figure 6A, exotoxin A was not detected in the colons of control mice, and only minimally detected in the colons of mice infected with non-treated PAO1, consistent with our observations in vitro. Moreover, the immunofluorescence appeared as distinct rod-like shapes, suggesting the exotoxin A was in intact *P. aeruginosa*. In contrast, abundant endotoxin A was detected in the colons of mice infected with zinc-exposed PAO1, and fluorescence was detected in both bacteria as well as in the tissue itself. Unfortunately, studies to quantify exotoxin A in the colon using immunoblotting were unsuccessful. Interestingly, and similar to what we observed in vitro, biofilm structures were detected only in the colons of mice infected with zinc-exposed PAO1 (Figure 6A). Finally, we found that while infection with non-treated PAO1 did not affect the expression of two key inflammatory cytokines, TNFα and IL-6, infection with zinc-exposed PAO1 significantly increased the expression of both TNFα (2-fold, *p* < 0.01) and IL-6 (3-fold, *p* < 0.01) (Figure 6B). This provides compelling evidence that the secretion of exotoxin A in response to excess zinc exposure is a key component of the commensal-to-pathogen transition in *P. aeruginosa*.

**Figure 7:**
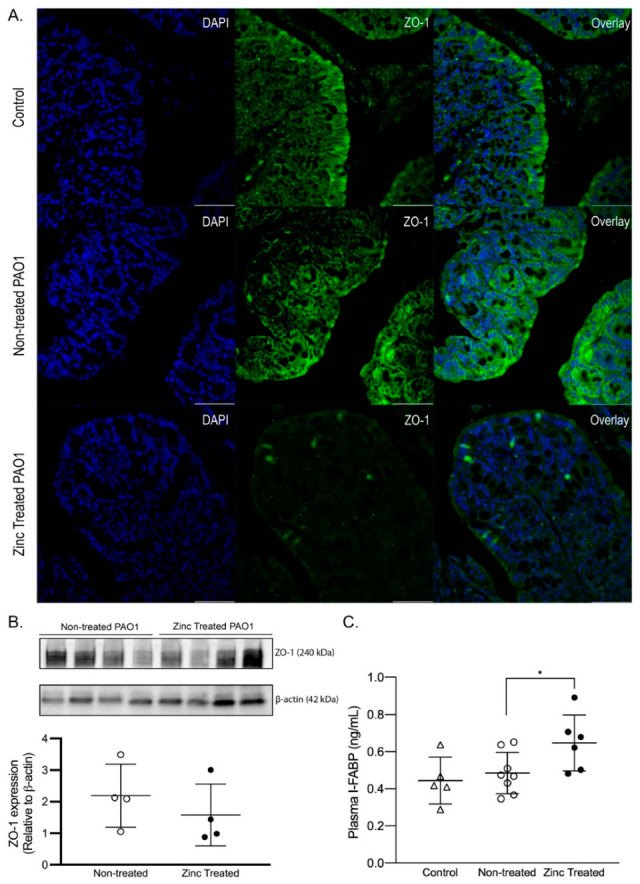
Zinc-exposed *P. aeruginosa* increased colon permeability. PAO1 was exposed to ZnSO_4_ (800 µM) for 16 h (Zinc treated) or left untreated (Non-treated) and orally gavaged in mice. Mice not gavaged with PAO1 were used for comparison (control). (**A**) Mice infected with zinc-exposed *P. aeruginosa* had disorganized ZO-1 localization in the colon. Representative images of ZO-1 (green) and nuclei stained with DAPI (blue) in the colons of control mice or mice infected with non-treated or zinc treated PAO1. Magnification, 40×; scale bar, 50 µm. (**B**) Zinc-exposed *P. aeruginosa* did not affect ZO-1 abundance in the colon. Representative immunoblots of ZO-1 in the colons of mice infected with non-treated or zinc treated PAO1. Membranes were stripped and re-probed for β-actin as a loading control. The data shown are from one representative experiment. The data represent the mean integrated optical density normalized to β-actin ± SD, *n* = 4 mice/group. The experiment was repeated twice, using four different mice/groups. (**C**) Zinc-exposed *P. aeruginosa* increased plasma I-FABP concentration. The data represent the mean concentration of I-FABP (ng/mL) ± SD, *n* = 5–8 mice/group. * *p* < 0.05, relative to non-treated PAO1 group, by ANOVA. Note that infection with non-treated PAO1 caused no increase in plasma I-FABP concentration.

Bacterial pathogens modulate intestinal tight junction structure and function to disrupt the intestinal barrier, which leads to both acute and chronic GI disorders. Since we showed excess zinc exposure increased expression of key virulence genes involved in motility and barrier dysfunction, we determined effects of zinc-exposed PAO1 on intestinal permeability by imaging the tight junction protein zonula occludens-1 (ZO-1) in the colon (Figure 7A) and quantifying intestinal fatty acid binding protein (I-FABP) in the plasma as an index of intestinal permeability (Figure 7B). In control mice, ZO-1 was clearly detected at the apical membrane in colonocytes. In contrast, in mice infected with non-treated PAO1, while some ZO-1 was detected on the villus tip, ZO-1 was also detected as a diffuse staining pattern throughout the colonocytes, suggesting that some ZO-1 was degraded and/or internalized. Finally, the apical membrane of colonocytes in mice infected with zinc-exposed PAO1 was completely devoid of ZO-1, instead ZO-1 was detected as distinct, punctate structures within the epithelial cells, suggesting major shifts in cellular distribution. To determine if differences in ZO-1 distribution was associated with reduced abundance, we measured ZO-1 using immunoblotting, but found no significant difference (Figure 7B). This suggests that excess zinc exposure in *P. aeruginosa* may trigger ZO-1 redistribution, but not necessarily degradation. Finally, mice infected with zinc-exposed PAO1 had significantly higher plasma I-FABP levels (Figure 7C, *p* < 0.05), collectively indicating increased motility of *P. aeruginosa* in promotes intestinal permeability, which is associated with reduced intestinal barrier function and illness.

## 3. Discussion

Recent studies highlight a role for *P. aeruginosa* in intestinal diseases [43,44], which necessitates the transition of this gut commensal to a pathogen. However, factors that drive commensal-to-pathogen transition and mechanism(s) responsible for this switch remain unclear. Herein, we demonstrate three principle findings: (1) physiologically relevant levels of excess zinc exposure broadly upregulated numerous virulence pathways in *P. aeruginosa* and promoted commensal-to-pathogen transition; (2) the zinc-dependent transcriptional regulator CzcR plays a critical role in the commensal-to-pathogen transition in response to excess zinc exposure; and (3) zinc-dependent activation of the commensal-to-pathogen transition leads to biofilm production, intestinal permeability, mucosal inflammation, and illness. To the best of our knowledge this is the first report to demonstrate that the exposure to physiologically relevant levels of a nutrient plays a molecular role in commensal-to-pathogen transition.

The Las QS system is a major regulatory node regulating over 10% of the genes in *P. aeruginosa*, including numerous virulence genes that are important for motility, colonization, biofilm formation, exotoxins, and antibiotic resistance [45]. Consistent with a previous report that showed physiologically relevant levels of zinc exposure increases the expression in the quorum sensing molecule lasI in *P. aeruginosa* [41], we found that excess zinc exposure more broadly and significantly upregulated the expression of the las QS system, including lasR and lasI, as well as the production and secretion of its autoinducer, 3-Oxo-C12HSL, which binds to lasR, triggering the broad expression of virulence factors such as elastase and exotoxins [46]. Moreover, zinc-dependent activation of the las QS system was associated with disrupted ZO-1 organization and intestinal architecture, which significantly increased intestinal permeability and illness in a pre-clinical mouse model. This is consistent with the role of lasI and 3-Oxo-C12HSL on the suppression and reorganization of tight junction proteins, causing the disruption of tight junction structures and greater intestinal permeability [10]. Architectural distortion and ZO-1 redistribution are considered the earliest sign of intestinal cell shedding, which may cause transient gaps or microerosions in epithelial barriers, leading to an increased intestinal permeability [47,48]. This is particularly important, as the intestinal barrier plays a key role in preventing the indiscriminate passage of intraluminal harmful substances to the vascular compartment, leading to a severe systemic inflammation [49,50]. Increased intestinal permeability can also induce bacterial translocation, leading to the enrichment of bacterial toxins in intestinal mucosa and systemic sepsis, causing chronic illness and even death [51]. While zinc is required for the pulmonary colonization of *Pseudomonas aeruginosa* in cystic fibrosis patients [52], to our knowledge a relationship between excess zinc intake and *Pseudomonas*-related GI illness has yet to be explored.

Biofilm development is closely interconnected to quorum sensing and physiological levels of excess zinc have been reported to induce biofilm formation in *P. aeruginosa* [35]. Here we showed both motility and biofilm formation were enhanced by excess zinc exposure due to the increased expression of type IV pili (pilB/D, fimV), flagella (filA), and rhamnolipids (rhlA/B), respectively. The upregulation of these factors provides P. aeruginosa with a greater ability to adhere to the epithelium, colonize and disrupt the microbiota in the host GI tract [53]. Similar to many other pathogens, internalization is the next essential step in *P. aeruginosa* infection [54]. Internalization protects *P. aeruginosa* from the host immune responses, and may lead to sepsis and severe organ damage [55,56]. Enhanced biofilm production and motility were associated with increased invasion capacity, which is consistent with the upregulation of genes important for type IV pili and flagella formation [57,58]. Type IV pili and flagella play a main role in seeking and locating appropriate host cells or finding a weak area within tissue for *P. aeruginosa* penetration and invasion [57]. Collectively, this suggests that excess zinc exposure may enhance the ability of *P. aeruginosa* to translocate to other organ systems and cause disease [59].

Once intracellular, *P. aeruginosa* releases lipopolysaccharide, which induces inflammation and necrosis [60]. In addition, exotoxin A is one of the most cytotoxic extracellular enzymes secreted by *P. aeruginosa* [61,62]. Exotoxin A is an NAD^+^-diphthamide-ADP-ribosyltransferase, which binds to specific receptors on host epithelial cells, leading to endocytosis [61]. Inside host cells, exotoxin A inhibits protein synthesis through ADP ribosylation of eukaryotic elongation factor 2, thereby activating necrosis and triggering broad inflammatory responses [62]. Similar to a previous report documenting the zinc-dependent activation of lasI through the zinc-dependent transcriptional regulator CzcR [41], we determined CzcR is also a critical regulator of toxA, thus expanding the role of CzcR in zinc-mediated pathogenesis. The profound increase in exotoxin A and the upregulation of pro-inflammatory cytokines such as TNFα and IL6 underpinned the cellular destruction we observed. This is consistent with observations in patients suffering from chronic intestinal inflammation who have exacerbated levels of TNF family cytokines in the intestinal tissue [63], and in mice where constitutive TNFα production leads to “Crohn’s like” inflammation in the small intestine [64]. Critically, zinc seems to act directly as a pathogenic switch in *P. aeruginosa*, as mice themselves were not fed a high zinc diet, and *P. aeruginosa* not exposed to excess zinc did not secrete exotoxin A or induce inflammation or illness. Intriguingly, a recent study indicates very high levels of zinc exposure (1000–5000 µM) promote antibiotic resistance in *P. aeruginosa* through robust activation of the AmgRS stress-responsive system; however, 200 µM appears sufficient to activate this system [65] supporting the need for further studies to probe the effects of physiologically relevant levels of excess zinc exposure on antibiotic resistance genes and risk for disease.

In conclusion, this study demonstrated that physiological levels of excess zinc exposure significantly and broadly promoted the commensal-to-pathogen transition and virulence of a gut opportunistic pathogen, *P. aeruginosa*, and provided evidence that these effects had important cellular and physiological consequences in the host GI tract. Further studies to understand the consequences of enhanced virulence in vitro using additional models and under different conditions are needed. Collectively, this suggests over-supplementation of this essential nutrient may have important negative consequences on GI health through the direct effects this has on modulating the gut microbiome. This may be particularly important in hospitalized individuals with underlying immune dysfunction or surgical disease.

## 4. Materials and Methods

### 4.1. Bacterial Strains and Zinc Treatment

PAO1 was purchased from American Type Culture Collection (#15692, ATCC; Manassas, VA, USA). *P. aeruginosa* containing a triple hemagglutinin tag on its czcR N-terminus (CzcR-HA PAO1) was kindly provided by Dr. Karl Perron (University of Geneva; Geneva, Switzerland) [41]. *P. aeruginosa* was cultured in LB (Alfa Aesar, #3339936; Haverhill, MA, USA) overnight at 200 rpm and 37 °C, then diluted to 5 × 10^7^ CFU/mL in LB (as a control) or LB containing zinc (800 µM, as ZnSO_4_, except for growth curve as indicated), and incubated for 16 h at 37 °C (shaking at 200 rpm). Zinc concentration was confirmed using Inductively Coupled Plasma-Optical Emission Spectrometer (ICP-OES; Agilent 5110; Agilent Technologies, Santa Clara, CA, USA) [66].

### 4.2. In Vitro Characterization

#### 4.2.1. *P. aeruginosa* Growth Curve

After overnight culture in LB, PAO1 was diluted to 0.05 × 10^9^ CFU/mL into 48-well plates in LB or LB containing zinc (100 µM to 2000 µM, as ZnSO_4_), and incubated for 16 h at 37 °C (shaking at 100 rpm). The optical density at 600 nm (OD_600_) was measured every 1 h by using a plate reader (SpectraMax M2; Molecular Devices; San Jose, CA, USA).

#### 4.2.2. *P. aeruginosa* Virulence Gene Expression

After 16 h zinc exposure, total RNA was extracted using TRIzol^™^ Max^™^ bacterial RNA isolation kit according to the manufacturer’s protocol (16096040; ThermoFisher; Waltham, MA, USA). RNA was quantified by Qubit^™^ Fluorometer and Qubit^™^ RNA BR Assay Kit (#Q10211; ThermoFisher; Waltham, MA, USA), and quality checked spectrophotometrically using a NanoDrop™ (#ND-2000; ThermoFisher; Waltham, MA, USA). Complementary DNA (cDNA) was generated from 1 µg of RNA, and 2 µL cDNA was used for qPCR. Iscript^™^ Reverse Transcription Supermix (#1708840, Bio-Rad; Hercules, CA, USA) and SsoAdvanced^™^ Universal SYBR^®®^ Green Supermix (#1725271, Bio-Rad; Hercules, CA, USA) were used for RT-qPCR using an RT-qPCR MyiQ^™^ 2 system (Bio-Rad; Hercules, CA, USA); threshold cycle was determined by Bio-Rad iQ^™^ 5 software. The melting curve and efficiency were evaluated for all primer pairs. The level of gene expression was calculated and normalized to *P. aeruginosa* sigma factor gene *rpoD* [67]. Primers (Table 1) were designed based on the *P. aeruginosa* gene database (http://www.pseudomonas.com/ accessed on 8 May 2019). All primers were purchased from ThermoFisher (Waltham, MA, USA).

#### 4.2.3. Quantitation of L-Homoserine Lactone (HSL)

HSL was measured using liquid chromatography tandem mass spectrometry in the positive electrospray ionization mode (LC-ESI-MS/MS) consisting of a Shimadzu LC-20AD chromatographic system coupled with an API 4000 triple quadruple mass spectrometer equipped with a Turbo Ion Spray source (Applied Biosystems; Waltham, MA, USA). Powder standards of 3-Oxo-C12HSL (#O9139) and C4-HSL-OH (#74359) with a certified purity of 98% and 96%, respectively, were obtained from Sigma Aldrich (Burlington, MA, USA). A standard curve containing both acyl-homoserine lactones was prepared by dissolving the standards in 35% methanol (#646377, Millipore Sigma; Burlington, MA, USA) and stored at −80℃ until analysis.

A PAO1 overnight culture was diluted to 0.05 CFU/mL in LB or 800 µM ZnSO_4_ (10 mL per tube, 9 samples/group), and incubated overnight at 37 °C. Bacterial supernatant was collected by centrifugation at 5000 g for 30 min at 4 °C and extracted three times with 5 mL dichloromethane [68]. Three extracts were combined and evaporated at 60 °C in a vacuum oven (SVAC2E, Sheldon Manufacturing; Cornelius, OR, USA), and the resultant residue was dissolved in 1 mL of 35% methanol. The ESI parameters and gas flow were optimized as follows: ion spray voltage was at 5000 V; the temperature of ion spray was set at 600 °C; nebulizer at 60 psi; heater/drying gas at 60 psi and curtain gas at 45 psi. Chromatographic separation was carried out on a Kinetex^®®^ C18 column, (4.6 × 150 mm, 2.6 µm particle size; Phenomenex; Washington, DC, USA) and column oven temperature was set at 30 °C. Sample injection volume was 10 μL. The mobile phases used were a mixture of methanol/water at 0.6 mL/min. The elution gradient was isocratic 35% methanol/ 65% water *v*/*v* for four min, linear gradient to 95% methanol/5% water for the next four min, followed by 3 min of post-column equilibration. The analytes were baseline separated from the sample matrix. Concentration was calculated using the external calibration curve. Quality control experiments included blanks, and cell culture media spiked with the standard cocktail at 10 ng/mL concentration. The analyte limits of detection, determined as three times the blank value, were 30 pg/mL (3-oxo-C12-HSL) and 50 pg/mL (C4-HSL). At the lowest standard of 0.2 ng/mL, the signal-to-noise ratio for each analyte was 7 (3-oxo-C12-HSL) and 6 (C4-HSL).

#### 4.2.4. Biofilm Assays

PAO1 overnight culture was diluted to 0.05 × 10^9^ CFU/mL in LB or LB containing 800 µM ZnSO_4_, and 400 µL was plated onto 48-well plates (*n* = 10 wells/group) and incubated overnight at 37 °C (without shaking). Following incubation, the cultures were removed from the wells, and the wells were washed three times with sterile PBS to remove the non-adherent bacteria. The adherent bacteria (*n* = 5 wells/group) were scraped, collected, and resuspended in 500 µL of PBS, then plated on LB-agar plates and incubated at 37 °C overnight. The adherent bacteria in the other five wells/groups were fixed with 500 µL of 75% ethanol for 15 min at room temperature. After removal of the ethanol, the plate was air-dried for 10 min, and biofilm was stained with 500 µL of BBL^™^ Gram Crystal Violet solution (#212525, BD Biosciences; Franklin Lakes, NJ, USA) for 10 min. The dye was removed, and the wells were washed with PBS to remove the excess unbound dye. The adherent biofilm was detached by 500 µL of 33% glacial acetic acid (#A38-500; ThermoFisher; Waltham, MA, USA). The absorbance of the resolubilized dye in each well was measure at 595 nm using a plate reader (SpectraMax M2; Molecular Devices; San Jose, CA, USA).

#### 4.2.5. Exotoxin A Quantitation

PAO1 overnight culture was diluted to 0.05 × 10^9^ CFU/mL in LB or LB containing 800 µM ZnSO_4_. After 16 h, 2 × 10^9^ CFU of zinc-treated or non-treated PAO1 bacterial culture was pelleted by centrifugation at 14,000× *g* for 60 s, and the medium was transferred and concentrated to 100 µL using a centrifugal filter with a 50 K molecular weight cut-off (UFC505024, Millipore Sigma; Burlington, MA, USA). 10 µL of each sample was separated on a 10% SDS-PAGE gel at 200 V for 1 h. Proteins were transferred to a nitrocellulose membrane at 100 V for 1 h. The membrane was blocked with 1% bovine serum albumin (BSA; #A9647, Millipore Sigma; Burlington, MA, USA) in 1× TBST (1× Tris-buffered saline, 0.1% Tween^®®^ 20; #P9416, Millipore Sigma; Burlington, MA, USA) at room temperature with rocking. After incubation with goat anti-exotoxin A antibody (1:1000; #LS-C50940, LifeSpan Biosciences; Seattle, WA, USA), the membranes were washed three times with 1× TBST and exotoxin A was detected using rabbit anti-goat IgG (1:10,000; #A16136; ThermoFisher; Waltham, MA, USA). Bands were visualized using SuperSignal^™^ West Femto Maximum Sensitivity Substrate (#34095; ThermoFisher; Waltham, MA, USA) and images were collected using the ChemiDoc^™^ MP imaging system (Bio-Rad; Hercules, CA, USA). Images were analyzed by Gel-Pro Analyzer software (version 4.0).

#### 4.2.6. ChIP Assays

ChIP was conducted as described [69] with modifications as follows: 2 × 10^10^ CFU of zinc-treated (800 µM ZnSO_4_) PAO1, non-treated PAO1, zinc-treated (800 µM ZnSO_4_) CzcR-HA PAO1, and non-treated CzcR-HA PAO1 were collected, resuspended in 20 mL LB containing 1.2% formaldehyde (#252549, Millipore Sigma; Burlington, MA, USA), and incubated at 37 °C for 10 min. Cross-linking was quenched by adding 1.375 M glycine/mL (#50046, Millipore Sigma; Burlington, MA, USA) of LB to reach a final concentration of 330 mM. Bacteria were collected by centrifugation at 5000 rpm for 10 min at 4 °C, washed twice with ice-cold Tris-buffered-saline, and resuspended in 1 mL B-PER bacterial protein extraction reagent (#78248; ThermoFisher; Waltham, MA, USA) containing lysozyme (#89833; ThermoFisher; Waltham, MA, USA) and protease inhibitor cocktail (#P8340, Millipore Sigma; Burlington, MA, USA). Sonication (output at 6 for 15 s, rest on ice for 1 min, repeated four times; XL-2000 series, Misonix Ultrasonic Liquid Processors; Alexandria, VA, USA) was performed to shear chromatin into 100–600 bp fragments. Soluble protein was collected by centrifugation at 14,000 rpm for 15 min at 4 °C. 50 µL of Pierce™ Anti-HA Magnetic Beads (88836; ThermoFisher; Waltham, MA, USA) was used to capture the CzcR-HA protein. Samples were eluted by heating at 65 °C for 5 h, and western blot was performed using an HA-tagged monoclonal antibody (1:1000; #32-6700; ThermoFisher; Waltham, MA, USA) to verify the successful immunoprecipitation of CzcR-HA protein. DNA was purified using the QIAquick^®®^ PCR purification kit (#28106, Qiagen; Germantown, MD, USA). 5 µL of purified DNA was used for PCR with Q5^®®^ high-fidelity DNA polymerase (#M0491S, New England BioLabs; Ipswich, MA, USA) and primers designed in the toxA promoter region (Table 1). PCR products were visualized by electrophoresis with a 1% agarose gel and imaged by the ChemiDoc^™^ MP imaging system (Bio-Rad; Hercules, CA, USA).

### 4.3. Caco-2 Co-Culture

#### 4.3.1. Cell Culture

Human colorectal adenocarcinoma cells (Caco-2 cells; ATCC, #HTB-37) were obtained commercially and cultured in DMEM (11965092; ThermoFisher; Waltham, MA, USA) supplemented with 10% Fetal Bovine Serum (#10082147; ThermoFisher; Waltham, MA, USA), 100 U/mL penicillin, 100 U/mL streptomycin (#15070063; ThermoFisher; Waltham, MA, USA) and 1% non-essential amino acids (#11140050; ThermoFisher; Waltham, MA, USA) at 37 °C in 5% CO_2_. Caco-2 cells were seeded in 24-well plates or 6.5 mm Transwell^®®^ inserts (#3413, Corning; Corning, NY, USA) at a density of 2.5 × 10^4^ cells/cm^2^.

PAO1 overnight cultures were obtained as previously described. Bacterial infection was previously described [70] with modifications as follows. Zinc-exposed and non-exposed PAO1 were collected and resuspended in fresh DMEM (without serum and antibiotics). Caco-2 cells were washed twice with fresh DMEM, and zinc-exposed or non-exposed PAO1 at 1 × 10^8^ CFU/cm^2^ were added. Cells were co-cultured with PAO1 at 37 °C in 5% CO_2_ until assayed, as described below.

#### 4.3.2. Invasion Assay

Caco-2 cells were seeded in 24-well plates and cultured until 21 d post-confluent. After Caco-2 monolayer was washed twice with fresh DMEM, 500 µL of zinc-exposed and non- exposed PAO1 in fresh DMEM (1 × 10^8^ CFU/cm^2^) were applied to Caco-2 monolayer, respectively, and 500 µL fresh DMEM was used as negative control. After 4 h incubation, medium and PAO1 were removed, and Caco-2 cells were washed with pre-warmed DMEM twice, then fresh DMEM containing 300 µg/mL gentamycin (GM, #345814-M, Millipore Sigma; Burlington, MA, USA) was added to cells and incubated for 1 h at 37 °C to kill any remaining extracellular PAO1. Caco-2 cells were washed with pre-warmed phosphate-buffered saline (PBS) three times to remove GM and dead PAO1. Caco-2 cells were lysed with 0.5% Triton^™^ X-100 (#X100, Millipore Sigma; Burlington, MA, USA) in LB to release intracellular PAO1. The cell lysates were plated on LB-agar plates and incubated at 37 °C overnight, and viable PAO1 were quantified by plate-counting.

#### 4.3.3. Permeability Assay

Caco-2 cells were seeded onto Transwell^®^ inserts, and Transepithelial resistance (TEER) was monitored every 2 days. Bacterial infection was conducted after TEER stabilization (~21 days post-confluence). Caco-2 cells were washed twice with pre-warmed DMEM twice and fresh DMEM was added to the basolateral chamber. 100 µL of zinc-exposed or non-exposed PAO1 (1 × 10^8^ CFU/cm^2^) were diluted into DMEM and fluorescein isothiocyanate-dextran (0.5 mg/mL;, #FD4, Millipore Sigma; Burlington, MA, USA) was added to the apical chamber. 100 µL LB with FD4 was added to another three apical chambers as negative controls. After 12 h at 37 °C, medium was collected from the basolateral chamber and the fluorescence (Ex-Max 490 nm/Em-Max 520 nm) was quantified as we previously described [71].

#### 4.3.4. LDH Cytotoxicity Assay

Caco-2 cells were seeded in 24-well plates and cultured until 21 d post-confluent and washed twice with DMEM. The culture medium (2 mL) from zinc-exposed and non-exposed PAO1 was collected and filtered (0.22 µm) to remove any bacteria. 50 µL filtrate was diluted 1:10 in DMEM (final volume 500 µL) and applied to each well of Caco-2 cells. 50 µL of LB containing 800 µM ZnSO_4_ was diluted 1:10 dilution in DMEM and then used as negative control. After 6 h at 37 °C, the Pierce LDH Cytotoxicity Assay kit (#88953, ThermoFisher; Waltham, MA, USA) was used to quantify lactate dehydrogenase (LDH) release into the medium per manufacturer’s instructions.

### 4.4. Preclinical Mouse Model

#### 4.4.1. Animals

The animal protocol was approved by the Institutional Animal Care and Use Committee (IACUC) at the University of Massachusetts Lowell. Male C57BL/6 mice aged ~five weeks old (*n* = 24) were purchased (Charles River Labs; Wilmington, MA, USA) and maintained on a 12-h day/night cycle under controlled temperature and humidity. All mice were fed a control powdered diet based on AIN93G (#96039620, MP Biomedicals; Irvine, CA, USA) containing adequate zinc (25 mg zinc/kg diet) for 3 weeks. At ~eight weeks of age, mice were fasted for 8 h and gavaged with PBS (negative control), 2 × 10^9^ CFU of zinc-treated PAO1, or 2 × 10^9^ CFU of non-treated PAO1 in PBS (*n* = 8/group) in a total volume of 0.2 mL per mouse using a soft-tipped flexible gavage tube (#50475764, Fisher Scientific; Waltham, MA, USA). Mice were weighed prior to infection, and body weight changes were measured daily through 10 d post-infection. Feces from zinc-treated and non-treated PAO1 mice were collected every 2 d into a sterile microfuge tube, suspended in 1 mL of sterile PBS, and plated on *P. aeruginosa*-selective cetrimide agar (#22470, Millipore Sigma; Burlington, MA, USA) plates to verify the colonization of PAO1. On d 10, mice were euthanized by CO_2_ asphyxiation and terminal exsanguination. Blood was drawn by cardiac puncture into heparinized tubes. Plasma was collected by centrifugation at 2000 rpm for 10 min and stored at −80 °C. Intestines and colons were dissected and perfused with sterile PBS. The distal one-third of the intestine (ileum) and entire colon were divided into three equal sections and stored as follows: proximal sections were snap-frozen and stored at −80 °C for protein analysis; the middle sections were stored in 1 mL of TRIzol^™^ (#15596018; ThermoFisher; Waltham, MA, USA) at −80 °C for RNA isolation; distal sections were fixed in phosphate-buffered paraformaldehyde (4%) overnight then stored in 70% ethanol at 4 °C until paraffin embedding.

#### 4.4.2. Tissue Staining and Immunofluorescence Imaging

Tissues were processed, embedded in paraffin, sectioned (5 µm), and stained with hematoxylin and eosin (H&E) as previously described [72]. Sections were used for IF imaging of *P. aeruginosa* exotoxin A and ZO-1 using the following antibodies: goat anti-exotoxin A (1:500; #LS-C50940, LifeSpan Biosciences; Seattle, WA, USA); donkey anti-goat AlexaFluor^®®^ 568 (1:1000; #A-11057, ThermoFisher; Waltham, MA, USA); rabbit anti-ZO-1 (1:100; #40-2200, ThermoFisher; Waltham, MA, USA); goat anti-rabbit AlexaFluor^®®^ 488 (1:1000; #A11008, ThermoFisher; Waltham, MA, USA). All sections were counterstained with 4′, 6-diamidino-2-phenylindole (DAPI, 175 µg/mL; #D1306, ThermoFisher; Waltham, MA, USA) to visualize nuclei. All images were captured using Leica Inverted Confocal Microscope SP8 (Leica Microsystems) and saved as .tif images.

#### 4.4.3. Illness Scoring

The illness score system by Wirtz and colleagues [42] was used and illness was assessed using the following parameters: daily body weight loss; general behavior; stool consistency; stool blood; and colon epithelial morphology and inflammatory cell infiltration documented using H&E staining.

#### 4.4.4. Immunoblotting

Colons were homogenized on ice in homogenization buffer (20 mM HEPES, 1 mM EDTA, 250 mM sucrose, and protease inhibitor, pH 7.4), then centrifuged at 5000× *g* for 10 min at 4 °C. The supernatant was collected, and protein concentration was quantified using the Qubit^®®^ protein assay (#Q33212, ThermoFisher; Waltham, MA, USA). 40 µg of each sample was used for immunoblotting as described above using anti-ZO-1 (1:1000; #40-2200, ThermoFisher; Waltham, MA, USA) then detected using goat anti-rabbit IgG (1:10,000; #31460, ThermoFisher; Waltham, MA, USA). Proteins were visualized using Femto (#4095, ThermoFisher; Waltham, MA, USA), imaged using the ChemiDoc^™^ MP imaging system (Bio-Rad; Hercules, CA, USA) and analyzed using the Gel-Pro Analyzer software (Version 4.0).

#### 4.4.5. Intestinal Permeability

Intestinal permeability was analyzed by quantifying the plasma levels of I-FABP using the mouse I-FABP ELISA kit (#LS-F21400, LifeSpan Biosciences; Seattle, WA, USA) according to the manufacturer’s protocol.

#### 4.4.6. Mouse Inflammatory Cytokine Gene Expression

Total RNA was extracted from colon using TRIzol™ Reagent according to the manufacturer’s protocol (#15596018, ThermoFisher; Waltham, MA, USA). The extracted RNA was quantified by Qubit^®®^ Fluorometer and Qubit^®®^ RNA BR Assay Kit (#Q10211, ThermoFisher; Waltham, MA, USA), and quality checked spectrophotometrically using a NanoDrop™ (#ND-2000, ThermoFisher; Waltham, MA, USA) and agarose gel electrophoresis. Complementary DNA (cDNA) was generated from 1 µg of RNA, and 2 µL cDNA was used for qPCR. Iscript^™^ Reverse Transcription Supermix (#1708840, Bio-Rad; Hercules, CA, USA) and SsoAdvanced^™^ Universal SYBR^®®^ Green Supermix (#1725271, Bio-Rad; Hercules, CA, USA) were used for RT-qPCR using an RT-qPCR MyiQ^™^ 2 system (Bio-Rad; Hercules, CA, USA). The threshold cycle was determined by Bio-Rad iQ^™^5 software. The melting curve and efficiency level were evaluated for all primer pairs. The level of gene expression was calculated and normalized to β-actin. Primers as listed in Table 1 were designed based on the National Center for Biotechnology Information (NCBI) gene database. All primers were purchased from ThermoFisher (Waltham, MA, USA).

### 4.5. Statistical Analysis

Data are presented as mean ± SD. Statistical analysis was performed by using students’ t-test (Mann–Whitney for data not normally distributed) or one-way ANOVA in GraphPad Prism software (version 8.3, GraphPad Software; San Diego, CA, USA) were noted, and significant differences were documented as *p* < 0.05.

## Figures and Tables

**Table 1:** List of amplicons and corresponding primers for RT-qPCR and ChIP.

Amplicon	Primer	Sequence (5′ to 3′)
*rpoD* (PA0576)	rpoD F	TGATCCAGGAAGGCAACATC
	rpoD R	GCGTAGGTGGAGAACTTGTAG
*lasR* (PA1430)	lasR F	AGAAGGAAGTGTTGCAGTGG
	lasR R	CTTCCGAGCAGTTGCAGATAA
*lasI* (PA1432)	lasI F	TGACGCACTCAGTCCTTATTAC
	lasI R	AGGTGTTCTTCAGCATGTAGG
*fimV* (PA3115)	fimV F	AAGTTCGTGACCTGGGTTC
	fimV R	TTCAGGTCGGTGAGGTAGTA
*pilB* (PA4526)	pilB F	TATCTCCGAACGACGCAAAC
	pilB R	GATCCGCATCACGATCTTCTC
*pilD* (PA4528)	pilD F	GCCTCATCGCCAACCATTT
	pilD R	AGCTTGAACAGCCAGAACAC
*toxA* (PA1148)	toxA F	TACCTGGGAAGGCAAGATCTAC
	toxA R	AATGCAGGCGATGACTGATG
*fliA* (PA1455)	fliA F	CAGCCTCAGTCACAACGAA
	fliA R	AATACAACGCCAGCACCA
*rhlA* (PA3479)	rhlA F	GCAGCTGGGACGAATACA
	rhlA R	GACTCCAGGTCGAGGAAATG
*rhlB* (PA3478)	rhlB F	GTCGGCGTTTCATGGAATTG
	rhlB R	TTCAGCCATCGAGCATCC
*czcR* (PA2523)	czcR F	CTGGGACATGAACTTCGACAA
	czcR R	AATGGTATGGATCAGCTTGAGG
*toxA* promoter	toxA Pro F	CCCTGCATGTATCCTCCGA
	toxA Pro R	GATGGCTCCTTTGATGGGTG
β-actin	β-actin F	AGGGAAATCGTGCGTGACAT
	β-actin R	GAACCGCTCGTTGCCAATAG
IL-6	IL-6 F	GATAAGCTGGAGTCACAGAAGG
	IL-6 R	TTGCCGAGTAGATCTCAAAGTG
TNF-α	TNF-α F	TTGTCTACTCCCAGGTTCTCT
	TNF-α R	GAGGTTGACTTTCTCCTGGTATG

## Data Availability

Data are contained within the article.
